# Modeling of Hydrogen-Charged Notched Tensile Tests of an X70 Pipeline Steel with a Hydrogen-Informed Gurson Model

**DOI:** 10.3390/ma16134839

**Published:** 2023-07-05

**Authors:** Robin Depraetere, Wim De Waele, Margo Cauwels, Tom Depover, Kim Verbeken, Stijn Hertelé

**Affiliations:** 1 Department of Electromechanical, Systems and Metal Engineering, Ghent University, Technologiepark-Zwijnaarde 46, 9052 Ghent, Belgium; stijn.hertele@ugent.be; 2 Department of Materials, Textiles and Chemical Engineering, Ghent University, Technologiepark-Zwijnaarde 46, 9052 Ghent, Belgium; margo.cauwels@ugent.be (M.C.); tom.depover@ugent.be (T.D.); kim.verbeken@ugent.be (K.V.)

**Keywords:** hydrogen embrittlement, pipeline steel, damage modelling, Gurson model, stress triaxiality, fracture locus

## Abstract

Hydrogen can degrade the mechanical properties of steel components, which is commonly referred to as “hydrogen embrittlement” (HE). Quantifying the effect of HE on the structural integrity of components and structures remains challenging. The authors investigated an X70 pipeline steel through uncharged and hydrogen-charged (notched) tensile tests. This paper presents a combination of experimental results and numerical simulations using a micro-mechanics-inspired damage model. Four specimen geometries and three hydrogen concentrations (including uncharged) were targeted, which allowed for the construction of a fracture locus that depended on the stress triaxiality and hydrogen concentration. The multi-physical finite element model includes hydrogen diffusion and damage on the basis of the complete Gurson model. Hydrogen-Assisted degradation was implemented through an acceleration of the void nucleation process, as supported by experimental observations. The damage parameters were determined through inverse analysis, and the numerical results were in good agreement with the experimental data. The presented model couples micro-mechanical with macro-mechanical results and makes it possible to evaluate the damage evolution during hydrogen-charged mechanical tests. In particular, the well-known ductility loss due to hydrogen was captured well in the form of embrittlement indices for the different geometries and hydrogen concentrations. The limitations of the damage model regarding the stress state are discussed in this paper.

## 1. Introduction

Hydrogen gas is an important energy carrier in the transition towards a low carbon economy [1]. Metal infrastructure has been considered for the storage and transport of hydrogen gas. However, the presence of hydrogen atoms in the steel can cause a loss in mechanical properties, such as ductility, fracture toughness, or fatigue resistance [2,3]. This phenomenon, known as ‘Hydrogen Embrittlement’ (HE), has to be analyzed carefully to avoid structural integrity issues leading to accidents.

Predicting fracture is of crucial importance when assessing structural integrity [4]. Continuum computational methods can be employed as an alternative to experimental testing [5,6]. These methods can describe damage development [7,8], replicate experimental test results, and support predictions of the structural behavior of structures with a complex geometry and/or loading conditions [9,10,11]. Additionally, considering the complexity of performing hydrogen-charged mechanical tests, a computational method for describing HE would be desirable. However, the accurate modelling of HE is difficult due to its complicated nature [12].

Several mechanisms have been set forth for explaining HE, which depend on the material microstructure and hydrogen concentration, among others [13,14]. These mechanisms can be classified into two groups—being predominantly brittle or plasticity-dominated. Hydrogen-Enhanced de-cohesion (HEDE) is a brittle mechanism arguing that hydrogen lowers the cohesive strength of the lattice [13]. The plasticity-dominated mechanisms, on the other hand, involve hydrogen-enhanced localized plasticity (HELP), which states that the dislocation mobility is enhanced by hydrogen [13,15], and hydrogen-enhanced strain-induced vacancies (HESIV) claim that hydrogen promotes vacancy formation [16]. A comprehensive overview by Djukic et al. [14] indicated that multiple HE mechanisms can act simultaneously. Despite the fact that the underlying mechanisms of HE are still under debate [14], there is ample evidence that the plasticity-dominated mechanisms play an important role in HE [15,17]. In 2019, Djukic et al. [14] stated that the occurrence of HELP was the greatest challenge in the analysis of the HE mechanisms. In a recent review article of the HELP mechanism, Martin et al. [15] highlighted the lack of predictive models as a crucial shortcoming. In summary, continuum modeling of the plasticity mechanisms is a crucial step towards the increased understanding of HE.

Next to the mechanistic effect of hydrogen, another key ingredient in the accurate modeling of HE is hydrogen diffusion, which is the transport of mobile hydrogen atoms through the steel lattice. This determines the spatial and temporal hydrogen concentration, which is responsible for the embrittlement. An important aspect of hydrogen diffusion is the increased diffusion towards regions of high hydrostatic stress, such as crack tips [18]. Previous studies have neglected this aspect in their continuum models [19,20,21], which is only acceptable when the loading rate is large enough compared to the diffusion coefficient [19].

The continuum modeling of plasticity-dominated mechanisms can be broadly divided into two categories: (i) fracture strain models and (ii) Gurson-type models. In fracture strain models, the fracture is controlled by the fracture locus, which is dependent on the stress triaxiality η, which is defined as the ratio of the hydrostatic to the equivalent stress. Damage accumulates upon plastic straining, and when this accumulation exceeds a critical value, the element loses its stiffness. Accelerated damage evolution due to hydrogen in these fracture strain models is incorporated through a reduction of the fracture locus with increasing hydrogen concentration [12,19,21,22,23]. Typically, only a single specimen geometry and, thus, a single stress triaxiality, is used for calibrating the degradation parameter [12,19,21,23]. Most authors introduce a multiplier of the fracture locus [21,22,23]; hence, the detrimental effect of hydrogen on the mechanical properties is independent of the stress triaxiality. However, multiple experimental studies suggest that embrittlement can increase for increasing stress triaxiality [24,25,26,27,28]. Moreover, hydrogen could change the failure mode, from internal necking coalescence to internal shearing coalescence at lower stress triaxialities [29]. Keeping these two effects in mind, using experimental results from hydrogen-charged tests in the lower stress triaxiality regime in order to predict HE at higher stress triaxialities might be questionable. An inherent advantage to the fracture strain approach is the simplicity associated with multiplying the fracture locus. A major drawback is that the method is phenomenological, which complicates the analysis of the damage processes during mechanical tests. Furthermore, the approach as presented in these studies [12,19,21,22,23] does not capture the gradual softening of the elements due to the accumulation of damage.

To overcome the limitations associated with simple fracture strain models, researchers have investigated the use of Gurson-type models for describing hydrogen degradation. These models are micro-mechanics-based, since they mathematically describe the full ductile failure process through void nucleation, growth, and coalescence. Nagumo [30] used the Gurson model with increasing void nucleation to model hydrogen-charged toughness tests, with the HESIV mechanism as the underlying theory. Yu et al. [31] proposed a ‘hydrogen-informed Gurson model’ where the void growth process was accelerated as a function of the local hydrogen concentration based on the HELP mechanism. Depraetere et al. [32] combined accelerated void nucleation and void growth, and they additionally introduced an acceleration in the void coalescence process, which was also based on the HELP mechanism. Finally, Lin et al. [33] showed the potential of an adapted Gurson model to include failure by decohesion according to the HEDE mechanism. It should be noted that Yu et al. [31], Depraetere et al. [32] and Lin et al. [33] only considered the development of the model, and did not apply the model yet to compare with experimental tests. Asadipoor et al. [20], on the other hand, employed a Gurson model for modeling hydrogen-charged flat strip tensile tests. However, only one geometry was modeled, and instead of making the Gurson parameters dependent on the hydrogen concentration, all Gurson parameters were simply recalibrated for the hydrogen-charged specimens. In other words, this approach is similar to the fracture strain approaches due to its phenomenological nature. Furthermore, since only a single geometry is investigated, the transferability of the obtained Gurson parameters to other geometries is uncertain.

In conclusion, all of the aforementioned studies concerning the continuum modeling of the plasticity-based mechanisms for accurately predicting HE lack at least one of the following three aspects. Firstly, some studies do not include hydrogen diffusion. Secondly, most studies consider a single geometry for determining the degradation parameters, thereby effectively assuming that HE is independent of the stress state. Thirdly, some studies make use of a phenomenological approach such as the fracture strain model, which complicates the connection with the physics observed during experiments.

The current work reports the experimental test data of an API 5L [34] X70 pipeline steel using four different geometries and three hydrogen concentrations (including uncharged specimens). Based on the macro-mechanical data and evidence of the failure mechanisms, a hydrogen-informed Gurson model was fitted. Finally, the hydrogen-affected fracture locus was analyzed to investigate the fracture process over a range of positive stress triaxialities.

## 2. Experimental Summary

### 2.1. Materials

The current study used the experimental test data of an API 5L [34] X70 pipeline steel. Test specimens originated from a pipeline that has been in use for approximately 30 years and features a banded ferrite–pearlite micro-structure with some degree of plastic anisotropy [28]. The chemical composition is given in Table 1. For a more detailed micro-structural characterization of the material, the reader is referred to Cauwels et al. [35].

**Figure 1:**
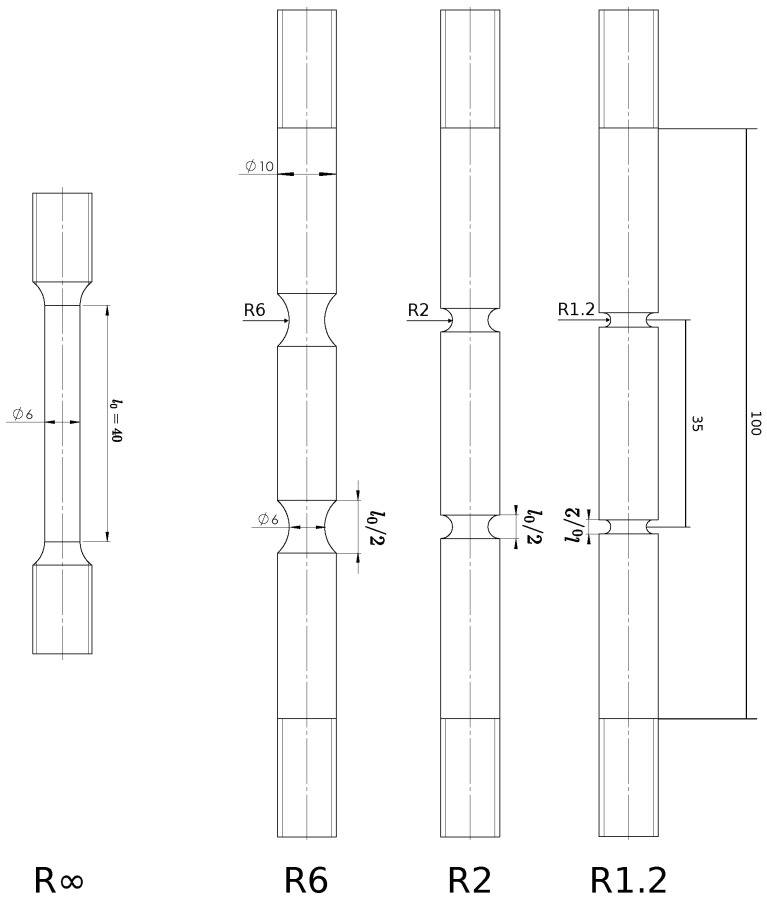
Specimen geometries tested. Dimensions in millimeters.

Four different tensile test geometries were extracted from the pipe along the longitudinal direction (also corresponding to the rolling direction of the steel), as shown in Figure 1. Both smooth (R∞) and double-notched (R6, R2, and R1.2) specimens were tested and covered a broad range of stress triaxialities. The specific levels of stress triaxiality associated with these geometries are discussed in Section 4. The use of two notches allowed for the post-mortem micro-scopic investigation of two separate deformation states: maximum force (considering the unfailed notch) and complete failure (considering the failed notch).

### 2.2. Experimental Methods

Prior to tensile testing, hydrogen charging was performed on a subset of the samples by submersion for 6 h in an electrolytic cell with a current density of 0.8 mA/cm^2^. Two different electrolytes were used, which resulted in two different nominal hydrogen concentrations $C_{L,0}$ after charging. The first electrolyte was 0.1 M NaOH with 1 g/L ${\mathrm{NH}}_{4}\mathrm{SCN}$, which resulted in $C_{L,0}=$ 0.36 ± 0.03 wppm. The second electrolyte was 0.5 M $H_{2}{\mathrm{SO}}_{4}$ with 1 g/L ${\mathrm{CH}}_{4}N_{2}S$, which resulted in $C_{L,0}=$ 1.09 ± 0.33 wppm. These concentrations were measured with hot extraction (G8 Galileo) and refer to the diffusible hydrogen concentrations.

After charging, the specimens were tensile tested at a fixed displacement rate. This rate was adapted for each geometry, such that the global strain rate in each specimen is equal to $2.5\;\times \;10^{-4}$ s^−1^. The reference length for the strain rate is the total ‘reduced area section length’, which is denoted as $l_{0}$ for each geometry on Figure 1. Note that the local strain rate in the double-notched specimens effectively doubles after maximum force, since all deformation will concentrate in the ‘weakest’ notch. The time between extracting the specimen from the electrolyte and starting the tensile test was approximately 7 min.

During the test, the lateral contractions of the specimen were monitored using two perpendicularly positioned cameras. This technique is also described in [28,36]. After post-processing, the diameters in both the transversal-to-rolling T direction $D_{T}$ and the through-thickness S direction $D_{S}$ were extracted. Consequently, the minimal instantaneous cross-sectional area was calculated as $A=\pi D_{T}D_{S}/4$. The advantage of this technique is that the strain values obtained are local strains at the minimal cross-section, rather than global strains averaged over the gauge length of an extensometer. Additionally, this enables the extraction of post-necking true stress versus true strain behavior [37]. Using the measured force *F* and the initial minimal cross-sectional area $A_{0}$, the true stress $\sigma_{t}$ and the true strain $\epsilon_{t}$ are calculated as:

(1) $$\sigma_{t}=F/A$$

(2) $$\epsilon_{t}=\ln\left(A_{0}/A\right).$$

Table 2 presents an overview of the experimental test data used for the calibration of the damage model parameters. Note that only a limited number of successful tests could be performed in the high triaxiality regime, since such a stress state leads to fracture of the micro-structural bands oriented along the S-direction, thus resulting in the occurrence of splits on the fracture surface that are accompanied by a sudden force drop [36]. The occurrence of a split is a stochastic event, and the cohesive strength of the interface between different layers is not included in the continuum HE model. Additionally, splitting is associated with a sudden drop of the stress triaxiality in the center. Therefore, these tests were not considered for the calibration of the model parameters and, thus, are not included in Table 2. In total, 14 uncharged tensile tests, 12 tensile tests with $C_{L,0}=$ 0.36 wppm, and 19 tests with $C_{L,0}=$ 1.09 wppm were successful.

### 2.3. Tensile Test Results

#### 2.3.1. Macro-Mechanical Responses

**Figure 2:**
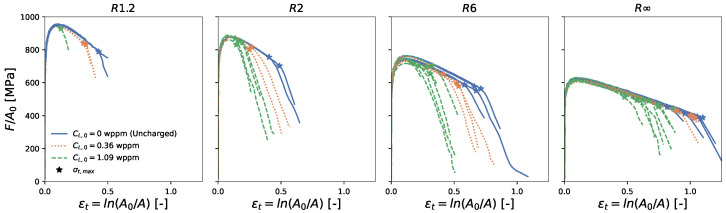
Normalized load $F/A_{0}$ versus true strain $\ln(A_{0}/A)$. The critical strain $\epsilon_{c}$ is indicated with a star.

**Figure 3:**
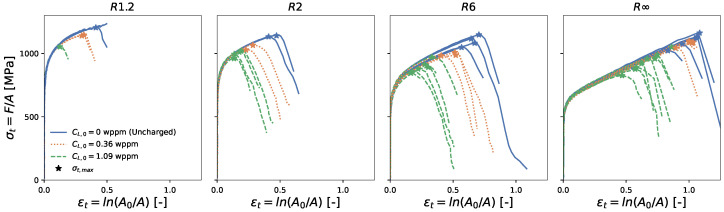
True stress F/A versus true strain $\ln(A_{0}/A)$. The critical strain $\epsilon_{c}$ is indicated with a star.

The resulting normalized load and true stress versus true strain curves are displayed in Figure 2 and Figure 3, respectively. The figures show that for all four geometries, the presence of hydrogen did not affect the strength. However, hydrogen did affect the ductility, with a larger hydrogen concentration leading to reduced fracture strains.

A point of interest is the true strain at the maximum true stress, since it is associated with the macro-crack initiation at the center of the specimen [6]. After this point, the load-carrying capacity rapidly drops, which can also be distinguished by a so-called ‘knee-point’ in the normalized load versus the plastic strain curve [38] (Figure 2). This point is further referred to as the critical strain $\epsilon_{c}$ and is marked with a star in Figure 2 and Figure 3.

An embrittlement index is commonly used for reflecting the amount of hydrogen-assisted degradation:

(3) $$EI\left[\%\right]=\frac{\epsilon_{c,air}-\epsilon_{c,H}}{\epsilon_{c,air}}*100.$$

Data of the observed maximum normalized load, critical strain, and embrittlement index are reported in Table 2. Note that, due to the limited number of tests for R1.2, it was impossible to determine a standard deviation for that specimen geometry. Overall, increasing embrittlement can be observed for increasing nominal hydrogen concentrations $C_{L,0}$ and increasing stress triaxiality, notwithstanding the exception of R1.2 with $C_{L,0}$ = 0.36 wppm. In particular, hydrogen-charged smooth round bars (R∞) show significantly lower degradation compared to the notched round bars, as determined by one-tailed independent *t*-tests (p<0.015 for all four combinations of R2 or R6 and for $C_{L,0}=$ 0.36 wppm or $C_{L,0}=$ 1.09 wppm).

#### 2.3.2. Failure Mechanisms

**Figure 4:**
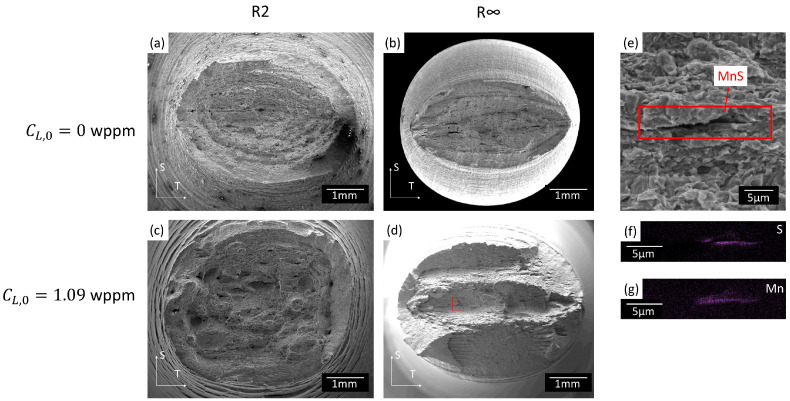
Scanning electron microscopy images of the fracture surfaces of an (**a**,**b**) uncharged and (**c**,**d**) hydrogen-charged ($C_{L,0}=$1.09 wppm) specimen with geometry R2 and R∞. (**e**) is a detail of (**d**) showing the presence of MnS inclusions. (**f**,**g**) is the energy-dispersive X-ray analysis (EDX) of the MnS inclusions in (**e**). Images (**a**–**d**) are shown at equal scale.

The failure mechanisms of the tested API 5L X70 pipeline steel without and with hydrogen are described in another study [28] and will be summarized here. Illustrative fracture surfaces of the R2 and R∞ geometry with $C_{L,0}=$ 0 wppm and $C_{L,0}=$ 1.09 wppm are provided in Figure 4. The uncharged tensile tests feature regular ductile fracture by micro-void coalescence. In the hydrogen-charged specimens, a mix of ductile dimples and quasi-cleavage was observed. The presence of MnS inclusions was observed on both uncharged and hydrogen-charged fracture surfaces (Figure 4e–g), thereby implying a significant role in the fracture process. From the fracture surfaces (Figure 4), it is clear that fractures in the lower stress triaxiality regime were increasingly dominated by shear failure. Moreover, the presence of hydrogen appeared to enhance the domination of shear-driven failure.

The damage processes were investigated in more detail using interrupted (notched) tensile tests and X-ray micro-CT [28] (The previous work [28] reported a hydrogen concentration of 0.89 wppm which corresponds to $C_{L,0}=$ 1.09 wppm in the present work. The difference is explained by a different method for measuring the hydrogen concentration, since the previous work used hot extraction at 300 ${}^{\circ }C$, whereas the current work used hot extraction at 900 ${}^{\circ }C$). Stress triaxiality levels ranging between 0.4 and 1.3 (averaged over the entire load–deformation trajectory) were tested using various geometries. The fracture processes in uncharged and hydrogen-charged specimens were observed to be similar, in the sense that they both consisted of void nucleation occurring in planes along the micro-structural bands, followed by void growth and void coalescence. However, two major differences were observed, which could have contributed to the reported ductility loss. First, the presence of hydrogen caused void nucleation to occur at much lower strains. While the minimal true strain at which voids were observed in uncharged smooth round bar samples was around 0.7, voids in hydrogen-charged samples could already be observed at strains of around 0.1. Second, hydrogen enhances lateral void growth, which reduces the cross-sectional ligament. However, this was not associated with an acceleration in the void volume growth. Both the accelerated void nucleation, as well as the enhanced lateral void growth, were present for all stress triaxiality levels tested. The reader is referred to Ref. [28] for more information.

## 3. Numerical Method

### 3.1. Hydrogen-Informed Gurson Model

The hydrogen-informed Gurson model is employed for modeling hydrogen-charged tensile tests. In this way, the ductile damage processes such as void nucleation, void growth, and void coalescence can be accelerated depending on the local lattice hydrogen concentration $C_{L}$, as postulated by the HELP and HESIV mechanisms. The diffusion of hydrogen is typically modeled using the derivation by Krom et al. [39]:

(4) $$\frac{C_{L}+C_{T}\left(1-C_{T}/\left(\alpha N_{T}\right)\right)}{C_{L}}\frac{\partial C_{L}}{\partial t}-\nabla \cdot \left(D_{L}\nabla C_{L}\right)+\nabla \cdot \left(\frac{D_{L}C_{L}{\bar{V}}_{H}}{RT}\nabla \sigma_{h}\right)+\frac{C_{T}}{N_{T}}\frac{dN_{T}}{d\epsilon_{p}}\frac{\partial \epsilon_{p}}{\partial t}=0,$$

which includes the hydrogen diffusion towards the hydrostatic stress gradient $\nabla \sigma_{h}$ and the division between the lattice hydrogen concentration $C_{L}$ and trapped hydrogen concentration $C_{T}$ (both in wppm). The lattice diffusion coefficient $D_{L}$ (in ${\mathrm{mm}}^{2}/s$) determines the rate of diffusion. Equation (4) also includes the equivalent plastic strain $\epsilon_{p}$ and the partial molar volume of hydrogen (${\bar{V}}_{H}$ = $2\;\times \;10^{-6}$ m^3^/mol). The equilibrium between $C_{L}$ and $C_{T}$ was postulated by Oriani [40] and is dependent on the trap binding energy $E_{B}$ (in kJ/mol):

(5) $$\frac{C_{T}/\left(\alpha N_{T}\right)}{1-C_{T}/\left(\alpha N_{T}\right)}=\frac{C_{L}/\left(\beta N_{L}\right)}{1-C_{L}/\left(\beta N_{L}\right)}\exp\left(-E_{B}/RT\right),$$

with $N_{L}$ and $N_{T}$ being the density of metal atoms and trapping sites, respectively, β being the number of interstitial sites per metal atom, and α being the number of sites per trap. A finite element implementation in Abaqus using user subroutines is provided in Depraetere et al. [32].

The evolution of damage is described by the complete Gurson model (CGM) [41], where the void volume fraction *f* is the damage indicator. Each element has an initial assigned void volume fraction $f_{0}$ and is completely fractured when reaching the final void volume fraction $f_{f}$. The yield criterion is given by the following:

(6) $$\phi \left(\sigma ,\bar{\sigma },f^{*}\right)={\left(\frac{\sigma_{e}}{\bar{\sigma }}\right)}^{2}+2q_{1}f^{*}\cosh\left(\frac{3q_{2}\sigma_{h}}{2\bar{\sigma }}\right)-1-q_{1}^{2}{f^{*}}^{2}=0,$$

with σ being the stress tensor, $\bar{\sigma }$ being the flow stress, $f^{*}$ being the effective void volume fraction, $\sigma_{e}$ being the von Mises stress, and $q_{1}$ and $q_{2}$ being empirical constants. Note that the effective void volume fraction $f^{*}$ is equal to the real void volume fraction *f* before coalescence, and it is accelerated upon coalescence.

The evolution of *f* is governed by the sum of void nucleation and void growth. Void nucleation is modeled using a strain-controlled approach based on a Weibull distribution:

(7) $$f_{nucleation}=f_{N}\left(1-\exp\left\{-{\left(\frac{\epsilon_{p}}{\epsilon_{N}}\right)}^{k}\right\}\right),$$

with k>0 being the shape parameter and $\epsilon_{N}>0$ being the scale parameter of the Weibull distribution, $f_{N}$ being the volume fraction of void nucleating particles, and $\epsilon_{p}$ being the equivalent plastic strain. Note that this is based on the well-known strain-controlled approach by Chu and Needleman [42], who used a Gaussian distribution. However, since a Weibull distribution produces strictly positive values, we regard this as a better choice for modeling void nucleation at reduced plastic strains [43]. For k>1, the scale parameter $\epsilon_{N}$ will approximate the mean value of the void nucleation distribution (both values differing by less than 10%) and serve a similar role as the mean nucleating strain $\epsilon_{N}$ in the approach by Chu and Needleman [42].

The void growth rate is derived from the volume conservation using the trace of the plastic strain rate tensor ${\dot{\epsilon }}_{kk}^{p}$ [41]. Void coalescence is based on the limit load criterion by Thomason [41].

Considering the desire for a micro-mechanics-inspired model for HE predictions, the Gurson parameters should be adapted to reflect hydrogen-assisted degradation based on the experimental observations of the failure mechanisms. An important aspect is that void nucleation in hydrogen-charged samples occurred at significantly reduced plastic strains. Accordingly, the mean void nucleation parameter $\epsilon_{N}$ was adapted for the presence of hydrogen. Data from several studies suggest that the degradation of the mechanical properties increases rapidly with increasing hydrogen concentration, after which saturation occurs [3,44]. For this reason, a degradation function was proposed that is exponentially decaying with increasing hydrogen concentration:

(8) $$\epsilon_{N}=\epsilon_{N,0}-\xi_{1}\left(1-\exp\left(-\xi_{2}*C_{L}\right)\right),$$

where $\epsilon_{N,0}$ equals the scale parameter without any hydrogen, and $\xi_{1}$ and $\xi_{2}$ are degradation parameters describing HE. It can be easily derived that $\epsilon_{N,0}-\xi_{1}$ represents the lowest possible value of $\epsilon_{N}$, while $\xi_{2}$ controls the speed of the degradation. Notably, Equation (8) only includes the lattice hydrogen concentration $C_{L}$, which disregards degradation due to the trapped hydrogen concentration $C_{T}$. This was done due to the lack of consensus as to whether numerical degradation models for hydrogen embrittlement should be based on $C_{L}$, on $C_{T}$, or on the sum of both [32,45]. Using Oriani’s equilibrium (Equation (5)), it can be derived that $C_{L}$ typically predominates over $C_{T}$ [45]. Accordingly, the current study assumes that $C_{L}$ is mainly responsible for hydrogen-related degradation.

Another micro-mechanical aspect observed in the experiments was the hydrogen enhanced lateral void growth. Since Gurson-Based models assume spherical void growth, void shape changes are neglected [5]. However, void shape effects may play a prominent role in the low stress triaxiality regime [6,46]. While extensions for including void shape and orientation changes exist [5,6,47], these models were deemed outside the scope of the present work.

Note that, when making the parameters $\epsilon_{N}$ dependent on the local lattice hydrogen concentration $C_{L}$, the nucleating distribution (Equation (7)) will change upon deformation due to hydrogen diffusion. For this reason, Equation (7) features the absolute nucleated voids $f_{nucleation}$ rather than the more common nucleation rate ${\dot{f}}_{nucleation}$. In this way, the number of voids nucleated at any point in time is a direct result of the instantaneous nucleating distribution. Additionally, it is specified that ${\dot{f}}_{nucleation}\geq 0$ such that nucleated voids cannot disappear.

### 3.2. Finite Element (FE) Models

**Figure 5:**
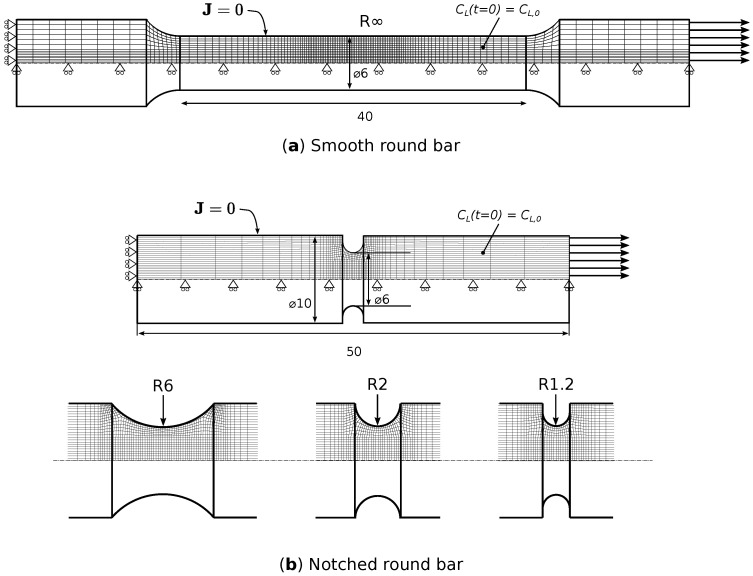
3D FE models for the (**a**) smooth round bar and (**b**) notched round bar tensile test simulations showing the geometry, the mesh, and the boundary conditions. Dimensions in millimeters.

Finite element (FE) models were constructed in Abaqus (2019) for simulating the (notched) uncharged and hydrogen-charged tensile tests (Figure 5). A quarter-circular cross-section was modeled with appropriate symmetry boundary conditions. Three-dimensional linear brick elements were employed (*C3D8T* in Abaqus). The mesh at the expected fracture zone consisted of cubic elements with a length of 0.2 mm. Note that only a single notch was modeled in Figure 5b, whereas the experiments featured double-notched round bars (Figure 1). To best represent the localized deformation in a single notch in the experiments, the initial displacement rate applied in the notched geometries resulted in a local strain rate of $1.25\;\times \;10^{-4}$ s^−1^ and was doubled when the maximum force was reached. For the smooth round bar, a constant strain rate of $2.5\;\times \;10^{-4}$ s^−1^ was used.

Given the predominance of $C_{L}$ over $C_{T}$ according to Equation (5), the specimens were given an initial lattice hydrogen concentration $C_{L,0}$ corresponding to the nominal diffusible hydrogen concentration after charging (Table 2). At the edge of the specimen, the hydrogen flux J was assumed to be zero, such that hydrogen remained inside the specimen. It should be noted that this is an idealization, and that setting the edge at zero is an alternative boundary condition to represent the contact with the atmosphere during the mechanical test [32,43]. However, a comparison of these two extreme boundary conditions resulted in only a minor difference (+/−10%) of the macro-mechanical response for the employed strain rate and diffusion coefficient. In other words, the hydrogen concentration at the center, where failure is expected to start, was only marginally affected by the hydrogen concentration at the edge for the performed tests. The effect of the adopted boundary condition is described in Appendix A.

**Figure 6:**
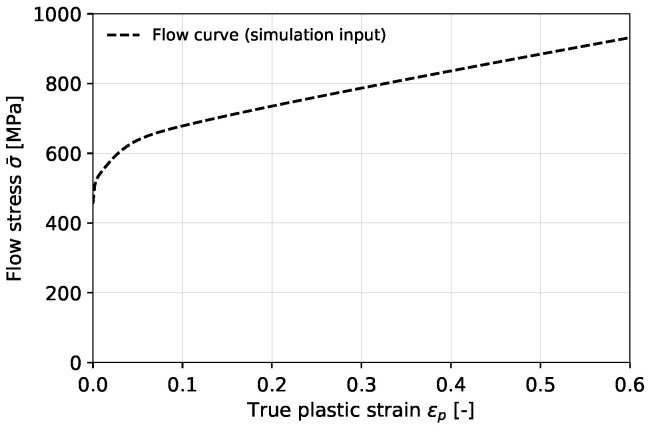
Flow stress $\bar{\sigma }$ versus true plastic strain $\epsilon_{p}$ of the API 5L X70 steel used as input for the simulations.

The hydrogen-related properties employed in the simulations can be found in Table 3. The diffusion coefficient $D_{L}$ was determined experimentally by permeation testing using a Devanathan–Stachursky setup, while the other parameters were adopted from literature [45,48]. The flow stress behavior of the API 5L X70 steel was determined as the true stress versus the true plastic strain obtained from a smooth round bar tensile test [43] and is presented in Figure 6. Note that this is an approximation, but it is frequently used for modeling ductile failure [49].

### 3.3. Calibration Procedure

The hydrogen-informed Gurson model contains several parameters that require calibration. The empirical constants $q_{1}$ and $q_{2}$ were determined using the tabulated values as a function of the yield strength and hardening coefficient from the unit cell approach by Faleskog et al. [50]. The remaining parameters ($f_{0}$, $f_{N}$, $f_{f}$, *k*, $\epsilon_{N,0}$, $\xi_{1}$, and $\xi_{2}$) were determined through inverse analysis by comparing the simulation output with experimental data, as explained below. This is a common approach for calibrating ductile damage models [8,51,52,53,54].

An objective function is defined based on the root mean squared error (RMSE) between the experimental and the numerical normalized load $F/A_{0}$ at equidistant levels of the true strain $\ln(A_{0}/A)$ for all four geometries. Note that the FE model does not take the observed plastic anisotropy into account, but an engineering approach is followed by comparing the plastic strains.

To properly reflect the adapted fracture behavior in the presence of hydrogen, the parameters $f_{0}$, $f_{N}$, $f_{f}$, *k*, and $\epsilon_{N,0}$ are first determined by minimizing the objective function using uncharged experimental data. Thereafter, using these calibrated parameters, the degradation parameters $\xi_{1}$ and $\xi_{2}$ are calibrated to best represent the hydrogen-charged tensile tests.

## 4. Results and Discussion

### 4.1. Agreement with Experiments

**Figure 7:**
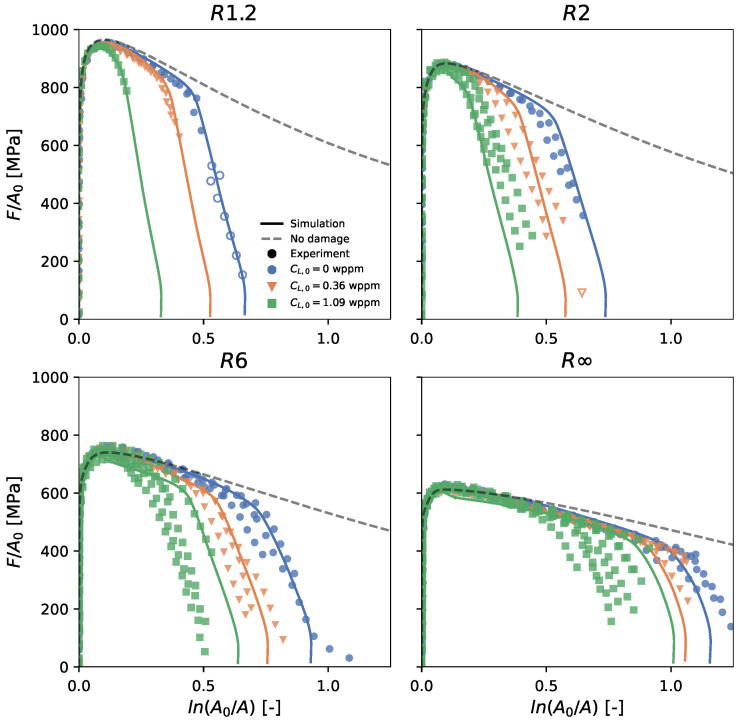
Comparison of the normalized load versus true strain curve between the experiments (symbols) and numerical simulations (lines). The simulation without damage is also shown. The colors refer to the different hydrogen concentrations.

After minimizing the objective function, the calibrated model parameters were obtained (Table 4). Figure 7 shows the comparison between the performed experiments and the predicted simulations. Note that only the filled markers were employed for computing the objective function, since the open markers correspond to a stress-strain state after a delamination occurrence. In general, an excellent agreement was found for all four geometries and three hydrogen concentrations. Only at higher hydrogen concentrations did the model overestimate the fracture strains in the lower stress triaxiality regime.

**Figure 8:**
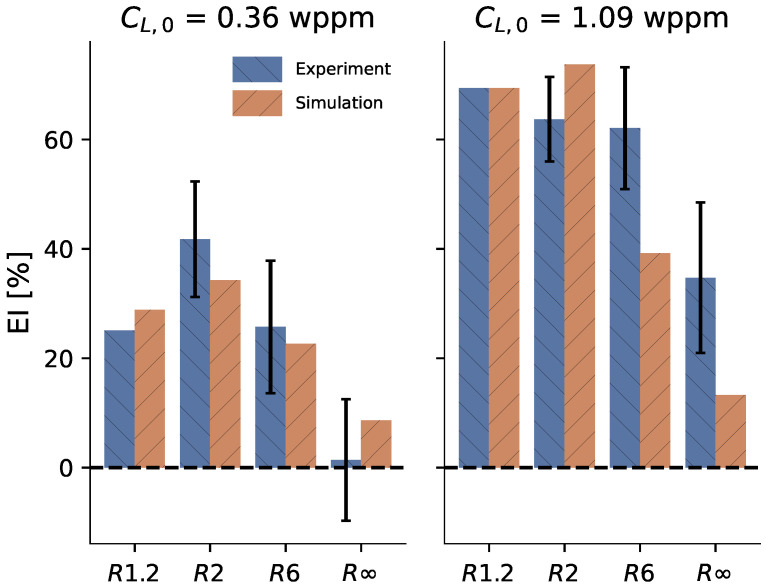
Comparison of the embrittlement indices (EI) on the critical strain $\epsilon_{c}$ from the experiments with the predictions by the HE model. The error bars represent the standard deviation of the quantity described by Equation (3). Note that, due to the limited amount of tests for R1.2, it was impossible to determine their standard deviation.

Similarly to the experiments, the ‘kneepoint’ can be extracted from simulations as the strain at the maximum true stress $\epsilon_{c}$. Using Equation (3), it can be transformed into an embrittlement index. Figure 8 compares the experimentally obtained embrittlement indices for each notch and geometry with the embrittlement indices from the simulations. In general, the model was capable of capturing the general trends of the hydrogen-assisted degradation quite well. Exceptions to this were the significantly overestimated fracture strains for the combination of the lowest considered stress triaxiality levels (R6, R∞) with the highest-considered hydrogen concentration ($C_{L,0}$ = 1.09 wppm).

We put forward two hypotheses to explain the poor agreement for the low triaxiality tests at higher hydrogen concentrations. A first hypothesis is the lack of an internal shearing coalescence criterion. The micromechanisms of void coalescence can be categorized into internal necking occurring at higher stress triaxialities and internal shearing at lower stress triaxialities [5,6,55,56]. Regarding the employed numerical damage model, Gurson-based models predicted the internal necking mechanism, and thus can yield poor numerical predictions when internal necking is not the dominant fracture mechanism [57,58,59,60]. Extensions, including the internal shearing coalescence, have been developed [47,61,62], but these typically require additional specific shear-dominated tests for calibration. Moreover, when modeling smooth round bar tensile tests with both coalescence mechanisms, it has been reported that internal shearing coalescence dominates [47,53]. Additionally, it is recognized that the presence of hydrogen can even change the mode from internal necking to internal shearing [29]. Micro-mechanical investigation of the fracture surfaces confirmed that failure was predominantly shear-driven in the low stress triaxiality regime and appeared to be enhanced by hydrogen (Figure 4). Accordingly, neglecting this increased shear-driven failure may lead to underestimated numerical predictions of the amount of embrittlement.

An additional hypothesis for the poor agreement is the omission of void shape effects in the hydrogen-informed Gurson model. Hydrogen enhances void growth perpendicular to the loading axis, hereby decreasing the remaining ligament area compared to the spherical void growth. Furthermore, it has been reported that void shape effects may be particularly important in the low stress triaxiality regime [6,46]. Therefore, a model that does not account for these void shape effects may lead to underestimated numerical predictions of the amount of embrittlement in this regime.

Both aspects should be addressed if the aim is to accurately model fracture in the low stress triaxiality regime.

**Figure 9:**
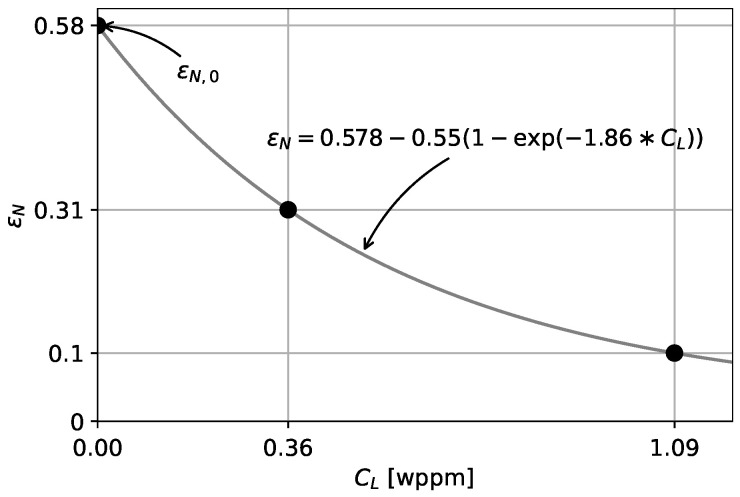
Degradation of the mean nucleating strain $\epsilon_{N}$ to best represent the hydrogen-charged tensile tests, as obtained from calibration.

The optimal degradation function of the mean nucleating strain $\epsilon_{N}$ (Equation (8)) is displayed in Figure 9. The mean nucleating strains for the nominal hydrogen concentrations tested were 0.31 and 0.1 for $C_{L,0}$ = 0.36 wppm and $C_{L,0}$ = 1.09 wppm respectively. Remarkably, $\epsilon_{N}$ for $C_{L,0}$ = 0 wppm and for $C_{L,0}$ = 1.09 wppm agreed well with the minimal strain at which voids were observed (around 0.7 and 0.1, respectively) using the X-ray micro-CT method that was described above [28]. This suggests a strong connection between the experiments and the hydrogen-informed Gurson model from a micromechanical point of view, since the model appeared to quantitatively capture the void nucleation mechanism.

### 4.2. Fracture Locus

An alternative method to investigate the effect of hydrogen and stress triaxiality on the fracture behavior of the API 5L X70 steel is through the fracture locus. From the FE simulations with the calibrated parameters (Table 4), an average triaxiality $\eta_{avg}$ can be extracted from the central element:

(9) $$\eta_{avg}=\frac{1}{\epsilon_{i}}\int_{0}^{\epsilon_{i}}\eta \;d\epsilon_{p},$$

where $\epsilon_{i}$ corresponds to the strain at which the central element coalesces according to Thomason’s limit criterion. The average triaxiality can be used to construct the fracture locus $\epsilon_{c}=f\left(\eta_{avg},C_{L,0}\right)$. Such a fracture locus is relevant, since it can be used as an input for other HE model predictions [12,19,21]. Note that, after the critical strain $\epsilon_{c}$ is reached, crack propagation occurs and the stress fields are disturbed. For this reason, the fracture locus was constructed using $\epsilon_{c}$, which is thought to be a better indicator of ductility than the fracture strain $\epsilon_{f}$ [6,38]. Additionally, it is well established that the fracture behavior in the lower stress triaxiality regime is affected not only by the stress triaxiality, but also by the Lode angle parameter that can be derived from the third stress invariant [63]. However, since the current study features only axisymmetric tensile experiments, which all have an identical Lode angle parameter, this effect was not investigated.

**Figure 10:**
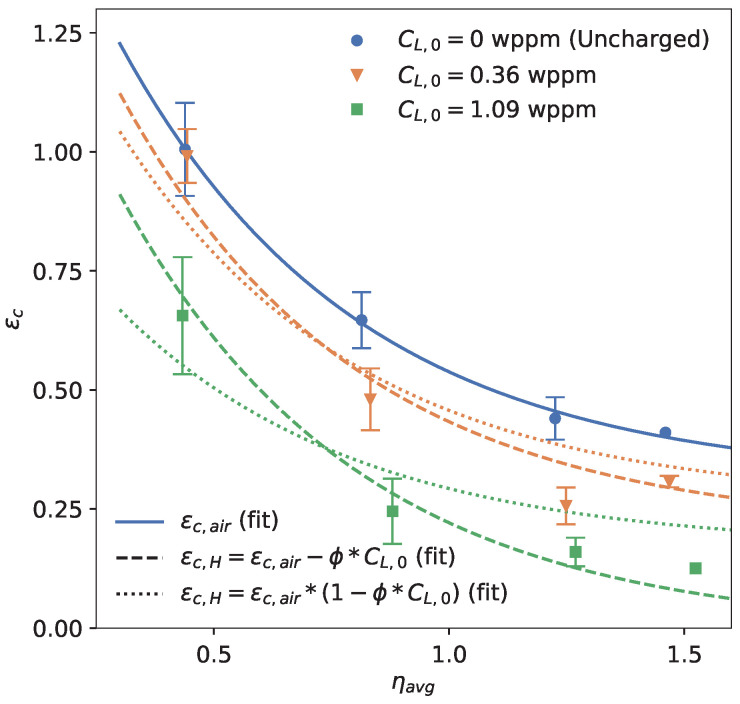
Fracture locus of the experiments, together with fitted lines. Error bars represent the standard deviation. The colors refer to the different hydrogen concentrations.

Figure 10 shows the resulting fracture locus. Note that the range of stress triaxiality values tested was roughly between 0.4 and 1.5. A common equation for expressing the fracture locus is as follows [27,64,65,66]:

(10) $$\epsilon_{c,air}=D_{1}\exp\left(-D_{2}*\eta_{avg}\right)+D_{3}.$$

The following was the best fit obtained using the averaged experimental data of each geometry from the uncharged tests and was added to Figure 10:

(11) $$\epsilon_{c,air}=1.67\exp\left(-1.98*\eta_{avg}\right)+0.31.$$

To include the effect of hydrogen in the fracture locus, a reduced fracture locus $\epsilon_{c,H}$ was considered. Two forms of degradation are presented in Table 5, which applied a subtraction and a multiplier to Equation (11). Both forms were fitted on the experimentally obtained $\epsilon_{c}$, which was averaged for each geometry and hydrogen concentration (Figure 10). The goodness of both fits was evaluated through the mean squared error MSE, which is found in Table 5.

From Figure 10 and the MSE, it is clear that a subtraction of the fracture locus better represented the performed experiments than a multiplication. A multiplication of the fracture locus corresponds to the EI being independent of stress triaxiality, which was not the case for these performed experiments (Table 2), as well as for other experimental studies [24,25,26,27]. Remarkably, a multiplication has been commonly assumed for degrading the fracture locus in the presence of hydrogen, without any experimental evidence [21,22,23]. It must be noted that Yu et al. [31] studied hydrogen degradation through unit cell simulations and derived a hydrogen-degraded failure locus that was best represented by a multiplication. However, they only considered internal necking failure and triaxiality values greater than 1. Moreover, their conclusions might have been significantly impacted by their assumptions regarding the modeling of degradation. We advise that particular attention must be given to the best representation of the triaxiality dependence of the hydrogen-affected fracture strains of a specific material.

## 5. Conclusions

The effect of hydrogen on the stress-triaxiality dependent fracture behavior of an API 5L X70 steel was studied from a combined experimental and numerical viewpoint. For this purpose, four different specimen geometries were tested at three different hydrogen concentrations (0, 0.36, and 1.09 wppm).

Hydrogen-assisted degradation appeared to increase with increasing stress triaxiality. Based on the plasticity-dominated HE mechanisms HELP and HESIV, a hydrogen-informed Gurson model was proposed for modeling ductile damage. The model parameters were fitted based on macro-mechanical deformations, as well as micro-mechanical evidence of the failure mechanisms. By reducing the void nucleating strain with increasing hydrogen concentration, good agreement between the experiments and the micro-mechanics inspired model predictions was obtained. Furthermore, the calibrated void nucleating strain agreed well with the experimental observations.

The predictions of the model significantly overestimated the fracture strains of the low stress triaxiality specimens. This was attributed to a lack in the hydrogen-informed Gurson model of either an internal shearing coalescence criterion or void shape effects capturing enhanced lateral void growth. While these issues should be addressed in future work, the presented model captured the experimental trends well within the higher stress triaxiality regime.

Finally, the effect of hydrogen on the fracture locus was investigated. It was shown that the effect of hydrogen on the triaxiality-dependent fracture strains was better represented by a subtraction, rather than a multiplication, of the uncharged fracture locus.

## Figures and Tables

**Table 1:** Average chemical composition of the investigated steel in weight percentage.

Material	C	Mn	Si	Cr	Ni	Nb	V	Mo	Cu	S	P	Fe
API 5L X70	0.108	1.633	0.426	0.030	0.022	0.054	0.068	0.002	0.018	0.003	0.015	Balance

**Table 2:** Numbers of tests for each geometry and nominal hydrogen concentration $C_{L,0}$, together with the average maximal normalized load ${\left(F/A_{0}\right)}_{max}$, the average critical strain $\epsilon_{c}$, and the average embrittlement index EI. When applicable, the standard deviation is also reported.

Notch	$C_{L,0}$ [wppm]	Number of Tests	${\left(F/A_{0}\right)}_{\max}$ [MPa]	$\epsilon_{c}$ [-]	EI [%]
R1.2	0	2 *	952 ± 6	0.41	
	0.36	2	944 ± 4	0.31 ± 0.01	25
	1.09	1	943	0.13	69
R2	0	2	879 ± 1	0.44 ± 0.04	
	0.36	2	876 ± 1	0.26 ± 0.04	42 ± 11
	1.09	4	876 ± 12	0.16 ± 0.03	64 ± 8
R6	0	4	751 ± 13	0.65 ± 0.06	
	0.36	3	740 ± 16	0.48 ± 0.06	26 ± 12
	1.09	6	743 ± 16	0.25 ± 0.07	62 ± 11
R∞	0	6	622 ± 4	1.01 ± 0.10	
	0.36	5	612 ± 6	0.99 ± 0.06	1 ± 11
	1.09	8	619 ± 8	0.66 ± 0.12	35 ± 14

* 1 sample splitted before reaching $\sigma_{t,max}$.

**Table 3:** Hydrogen-Related material properties used for the FE simulations.

$D_{L}$ [mm^2^/s]	$E_{B}$ [kJ/mol]	$N_{L}$ [m^−3^]	$N_{T}$ [m^−3^]	α	β	*T* [K]
4.5 × 10^−4^	−30	8.47 × 10^28^	$10^{23.26-2.33\exp(-5.5\epsilon_{p})}$	1	6	300

**Table 4:** Calibrated damage parameters for the investigated API 5L X70 steel.

$q_{1}$	$q_{2}$	$f_{0}$	$f_{N}$	$f_{f}$	*k*	$\epsilon_{N,0}$	$\xi_{1}$	$\xi_{2}$
1.42	0.96	0.0034	0.031	0.43	6	0.578	0.55	1.86

**Table 5:** Two types of degradation functions for representing the fracture locus.

$\epsilon_{c,H}$	ϕ	MSE
$\epsilon_{c,air}-\phi *C_{L,0}$	0.29	0.0032
$\epsilon_{c,air}\left(1-\phi *C_{L,0}\right)$	0.42	0.0089

**Figure A1:**
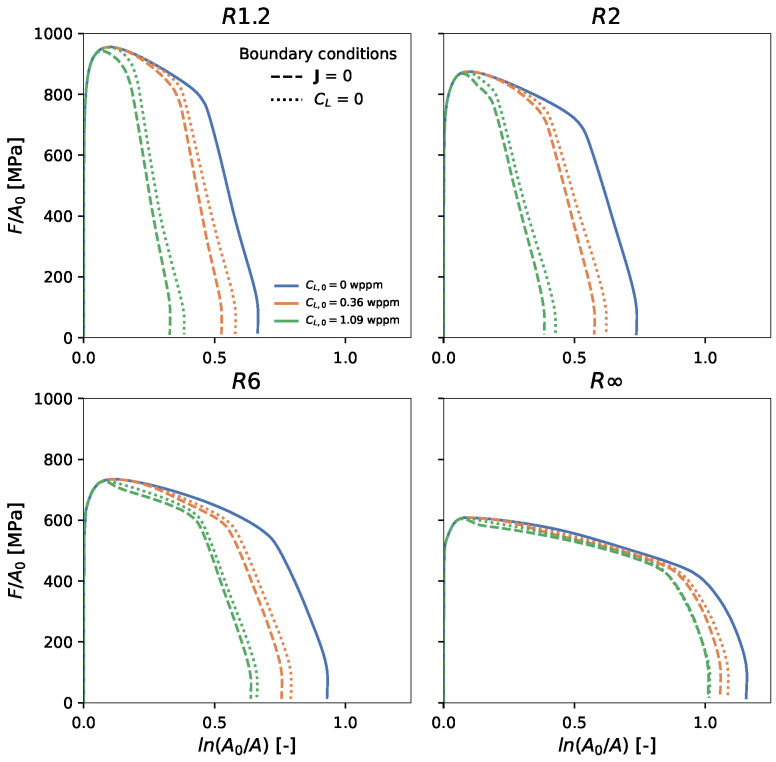
Comparison of the normalized load versus the true strain curve for the two extreme boundary conditions. The colors correspond to the colors used in Figure 7.

**Figure A2:**
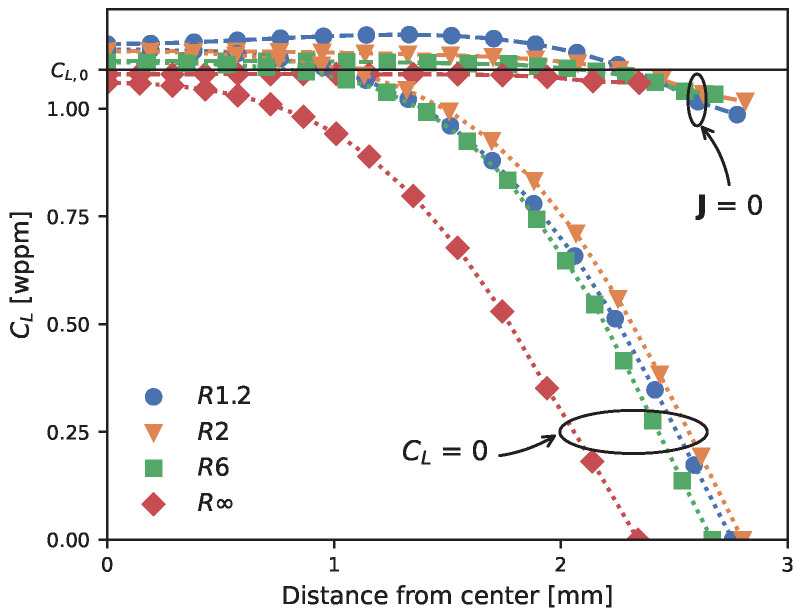
Hydrogen concentration $C_{L}$ versus the distance from the center. Only the simulations with $C_{L,0}$ = 1.09 wppm are shown.

## Data Availability

The experimental stress-strain curves (see Figure 2 and Figure 3) are available at [67].

## Appendix A. Effect of Hydrogen-Related Boundary Condition on Results

The boundary condition proposed in Section 3.2 assumes that the hydrogen flux J is zero at the edge. The alternative boundary condition specifying $C_{L}$ = 0 at the edge describes an idealized opposite scenario where the hydrogen concentration is diffusing out of the specimen. An accurate representation of the conditions at the surface should be comprised between these two extremes [68]. To investigate the effect hereof on the obtained results, the simulations were repeated with boundary condition $C_{L}$ = 0. The damage parameters from Table 4 were employed, which were derived after calibration using simulations with the boundary condition J = 0. The resulting normalized load versus the true strain curves for each geometry are shown in Figure A1. By specifying $C_{L}$ = 0, increased fracture strains were predicted due to the decreased hydrogen concentrations. While the effect increases for increased stress triaxiality, only a minor difference was observed between both extreme boundary conditions.

The distribution of hydrogen for the different geometries and boundary conditions is shown in Figure A2. The data were obtained from the simulations with an initial hydrogen concentration $C_{L,0}$ = 1.09 wppm at the moment where the central element coalesced according to Thomason’s limit criterion. The figure illustrates that the choice of boundary condition has a minimal impact on the hydrogen concentration in the center of the specimen, which is the location where failure initiates. This explains the limited difference in the normalized load versus the true strain curves (Figure A1). It is noted that the boundary conditions will play a larger role when either the strain rate is decreased, the diffusion coefficient is increased, or the fracture initiates at the outside, e.g., during a fracture toughness test.

## References

[1] European Commission A Hydrogen Strategy for a Climate-Neutral Europe (52020DC0301); Technical Report; European Commission 2020. https://eur-lex.europa.eu/legal-content/EN/TXT/?uri=CELEX:52020DC0301.

[2] Johnson W. (1875). On some remarkable changes produced in iron and steel by the action of hydrogen and acids. Proc. R. Soc. Lond..

[3] Laureys A., Depraetere R., Cauwels M., Depover T., Hertelé S., Verbeken K. (2022). Use of existing steel pipeline infrastructure for gaseous hydrogen storage and transport: A review of factors affecting hydrogen induced degradation. J. Nat. Gas Sci. Eng..

[4] Traidia A., Chatzidouros E.V., Jouiad M. (2018). Review of hydrogen-assisted cracking models for application to service lifetime prediction and challenges in the oil and gas industry. Corros. Rev..

[5] Besson J. (2010). Continuum Models of Ductile Fracture: A Review. Int. J. Damage Mech..

[6] Pineau A., Benzerga A.A., Pardoen T. (2016). Failure of metals I: Brittle and ductile fracture. Acta Mater..

[7] Boyce B.L., Kramer S.L., Bosiljevac T.R., Corona E., Moore J.A., Elkhodary K., Simha C.H., Williams B.W., Cerrone A.R., Nonn A. (2016). The second Sandia Fracture Challenge: Predictions of ductile failure under quasi-static and moderate-rate dynamic loading. Int. J. Fract..

[8] Paredes M., Wierzbicki T., Zelenak P. (2016). Prediction of crack initiation and propagation in X70 pipeline steels. Eng. Fract. Mech..

[9] Nonn A., Kalwa C. (2012). Simulation of ductile crack propagation in high-strength pipeline steel using damage models. Proc. Bienn. Int. Pipeline Conf. IPC.

[10] Kristensen P.K., Niordson C.F., Martínez-Pañeda E. (2020). Applications of phase field fracture in modelling hydrogen assisted failures. Theor. Appl. Fract. Mech..

[11] Brinnel V., Schaffrath S., Münstermann S., Feldmann M. (2020). Efficient, scale-bridging simulation of ductile failure in a burst test using damage mechanics. Int. J. Press. Vessel. Pip..

[12] Huang S., Hui H. (2022). Predictive environmental hydrogen embrittlement on fracture toughness of commercial ferritic steels with hydrogen-modified fracture strain model. Int. J. Hydrogen Energy.

[13] Lynch S.P. (2011). Hydrogen embrittlement (HE) phenomena and mechanisms. Stress Corrosion Cracking: Theory and Practice.

[14] Djukic M.B., Bakic G.M., Sijacki Zeravcic V., Sedmak A., Rajicic B. (2019). The synergistic action and interplay of hydrogen embrittlement mechanisms in steels and iron: Localized plasticity and decohesion. Eng. Fract. Mech..

[15] Martin M.L., Dadfarnia M., Nagao A., Wang S., Sofronis P. (2019). Enumeration of the hydrogen-enhanced localized plasticity mechanism for hydrogen embrittlement in structural materials. Acta Mater..

[16] Nagumo M., Takai K. (2019). The predominant role of strain-induced vacancies in hydrogen embrittlement of steels: Overview. Acta Mater..

[17] Robertson I.M., Sofronis P., Nagao A., Martin M.L., Wang S., Gross D.W., Nygren K.E. (2015). Hydrogen Embrittlement Understood. Metall. Mater. Trans. Process. Metall. Mater. Process. Sci..

[18] Martínez-Pañeda E., Del Busto S., Niordson C.F., Betegón C. (2016). Strain gradient plasticity modeling of hydrogen diffusion to the crack tip. Int. J. Hydrogen Energy.

[19] Huang S., Zhang Y., Yang C., Hu H. (2020). Fracture strain model for hydrogen embrittlement based on hydrogen enhanced localized plasticity mechanism. Int. J. Hydrogen Energy.

[20] Asadipoor M., Kadkhodapour J., Pourkamali Anaraki A., Sharifi S.M., Darabi A.C., Barnoush A. (2020). Experimental and Numerical Investigation of Hydrogen Embrittlement Effect on Microdamage Evolution of Advanced High-Strength Dual-Phase Steel. Met. Mater. Int..

[21] Youn G.G., Kim Y.J., Kim J.S., Lam P.S. (2021). A Fracture Strain Based Numerical Prediction Method for Hydrogen Effect on Fracture Toughness. Int. J. Mech. Sci..

[22] Pfuff M., Dietzel W. Mesoscale modeling of hydrogen assisted crack growth in heterogeneous materials. Proceedings of the 11th International Conference on Fracture 2005, ICF11.

[23] Kim N.H., Oh C.S., Kim Y.J., Yoon K.B., Ma Y.W. (2012). Hydrogen-assisted stress corrosion cracking simulation using the stress-modified fracture strain model. J. Mech. Sci. Technol..

[24] Kwon D.I., Asaro R.J. (1990). Hydrogen-assisted ductile fracture in spheroidized 1518 steel. Acta Metall. Mater..

[25] Cayón A., Gutiérrez-Solana F., Arroyo B., Álvarez J. (2020). Hydrogen Embrittlement Processes in Microalloyed Steel Notched Tensile Samples. Theor. Appl. Fract. Mech..

[26] Nguyen T.T., Tak N., Park J., Nahm S.H., Beak U.B. (2020). Hydrogen embrittlement susceptibility of X70 pipeline steel weld under a low partial hydrogen environment. Int. J. Hydrogen Energy.

[27] Bal B., Çetin B., Bayram F.C., Billur E. (2020). Effect of hydrogen on fracture locus of Fe–16Mn–0.6C–2.15Al TWIP steel. Int. J. Hydrogen Energy.

[28] Depraetere R., De Waele W., Cauwels M., Depover T., Verbeken K., Boone M., Hertelé S. (2023). Influence of stress triaxiality on hydrogen assisted ductile damage in an X70 pipeline steel. Mater. Sci. Eng. A.

[29] Yu H., Olsen J.S., He J., Zhang Z. (2018). Hydrogen-microvoid interactions at continuum scale. Int. J. Hydrogen Energy.

[30] Nagumo M. (2004). Hydrogen related failure of steels - A new aspect. Mater. Sci. Technol..

[31] Yu H., Olsen J.S., Alvaro A., Qiao L., He J., Zhang Z. (2019). Hydrogen informed Gurson model for hydrogen embrittlement simulation. Eng. Fract. Mech..

[32] Depraetere R., De Waele W., Hertelé S. (2021). Fully-coupled continuum damage model for simulation of plasticity dominated hydrogen embrittlement mechanisms. Comput. Mater. Sci..

[33] Lin M., Yu H., Ding Y., Wang G., Olden V., Alvaro A., He J., Zhang Z. (2022). A predictive model unifying hydrogen enhanced plasticity and decohesion. Scr. Mater..

[34] American Petroleum Institute (2018). API Specification 5L—Line Pipe.

[35] Cauwels M., Depraetere R., De Waele W., Hertelé S., Depover T., Verbeken K. (2022). Influence of electrochemical hydrogenation parameters on microstructures prone to hydrogen-induced cracking. J. Nat. Gas Sci. Eng..

[36] Depraetere R., Cauwels M., de Waele W., Depover T., Verbeken K., Hertelé S. (2020). Calibrating a ductile damage model for two pipeline steels: Method and challenges. Procedia Struct. Integr..

[37] Shokeir Z., Besson J., Belhadj C., Petit T., Madi Y. (2022). Edge tracing technique to study post-necking behavior and failure in Al alloys and anisotropic plasticity in line pipe steels. Fatigue Fract. Eng. Mater. Struct..

[38] Basu S., Benzerga A.A. (2015). On the path-dependence of the fracture locus in ductile materials: Experiments. Int. J. Solids Struct..

[39] Krom A.H., Koers R.W., Bakker A. (1999). Hydrogen transport near a blunting crack tip. J. Mech. Phys. Solids.

[40] Oriani R.A. (1970). The diffusion and trapping of hydrogen in steel. Acta Metall..

[41] Zhang Z.L., Thaulow C., Ødegård J. (2000). Complete Gurson model approach for ductile fracture. Eng. Fract. Mech..

[42] Chu C.C., Needleman A. (1980). Void nucleation effects in biaxially stretched sheets. J. Eng. Mater. Technol. Trans. ASME.

[43] Depraetere R., Cauwels M., De Waele W., Depover T., Verbeken K., Hertelé S. Single-edge notched tension testing for assessing hydrogen embrittlement: A numerical study of test parameter influences. Proceedings of the ECCOMAS Congress 2022—8th European Congress on Computational Methods in Applied Sciences and Engineering. CIMNE.

[44] Xu K., Gangloff R., Somerday B. (2012). Hydrogen embrittlement of carbon steels and their welds. Gaseous Hydrogen Embrittlement of Materials in Energy Technologies, Volume 1: The Problem, Its Characterisation and Effects on Particular Alloy Classes.

[45] Jemblie L., Olden V., Akselsen O.M. (2017). A coupled diffusion and cohesive zone modelling approach for numerically assessing hydrogen embrittlement of steel structures. Int. J. Hydrogen Energy.

[46] Khan I.A., Srivastava A., Needleman A., Benzerga A.A. (2021). An analysis of deformation and failure in rectangular tensile bars accounting for void shape changes. Int. J. Fract..

[47] Nguyen V.D., Pardoen T., Noels L. (2020). A nonlocal approach of ductile failure incorporating void growth, internal necking, and shear dominated coalescence mechanisms. J. Mech. Phys. Solids.

[48] Kumnick A.J., Johnson H.H. (1980). Deep trapping states for hydrogen in deformed iron. Acta Metall..

[49] Mirone G. (2004). A new model for the elastoplastic characterization and the stress-strain determination on the necking section of a tensile specimen. Int. J. Solids Struct..

[50] Faleskog J., Gao X., Fong Shih C. (1998). Cell model for nonlinear fracture analysis—I. Micromechanics calibration. Int. J. Fract..

[51] Springmann M., Kuna M. (2005). Identification of material parameters of the Gurson-Tvergaard-Needleman model by combined experimental and numerical techniques. Comput. Mater. Sci..

[52] Broggiato G.B., Campana F., Cortese L. (2007). Identification of material damage model parameters: An inverse approach using digital image processing. Meccanica.

[53] Achouri M., Germain G., Dal Santo P., Saidane D. (2013). Experimental characterization and numerical modeling of micromechanical damage under different stress states. Mater. Des..

[54] Zhang T., Lu K., Mano A., Yamaguchi Y., Katsuyama J., Li Y. (2021). A novel method to uniquely determine the parameters in Gurson–Tvergaard–Needleman model. Fatigue Fract. Eng. Mater. Struct..

[55] Bao Y., Wierzbicki T. (2004). On fracture locus in the equivalent strain and stress triaxiality space. Int. J. Mech. Sci..

[56] Danas K., Ponte Castañeda P. (2012). Influence of the Lode parameter and the stress triaxiality on the failure of elasto-plastic porous materials. Int. J. Solids Struct..

[57] Reis F.J., Malcher L., Pires F.M., De Sá J.M. (2010). A comparison of shear mechanisms for the prediction of ductile failure under low stress triaxiality. Int. J. Struct. Integr..

[58] Li H., Fu M.W., Lu J., Yang H. (2011). Ductile fracture: Experiments and computations. Int. J. Plast..

[59] Kiran R., Khandelwal K. (2014). Gurson model parameters for ductile fracture simulation in ASTM A992 steels. Fatigue Fract. Eng. Mater. Struct..

[60] Cao T.S., Mazière M., Danas K., Besson J. (2015). A model for ductile damage prediction at low stress triaxialities incorporating void shape change and void rotation. Int. J. Solids Struct..

[61] Nahshon K., Hutchinson J.W. (2008). Modification of the Gurson Model for shear failure. Eur. J. Mech. A/Solids.

[62] Xue L. (2008). Constitutive modeling of void shearing effect in ductile fracture of porous materials. Eng. Fract. Mech..

[63] Bai Y., Wierzbicki T. (2008). A new model of metal plasticity and fracture with pressure and Lode dependence. Int. J. Plast..

[64] Johnson G.R., Cook W.H. (1985). Fracture characteristics of three metals subjected to various strains, strain rates, temperatures and pressures. Eng. Fract. Mech..

[65] Münstermann S., Schruff C., Lian J., Döbereiner B., Brinnel V., Wu B. (2013). Predicting lower bound damage curves for high-strength low-alloy steels. Fatigue Fract. Eng. Mater. Struct..

[66] Bergo S., Morin D., Børvik T., Hopperstad O.S. (2020). Micromechanics-based identification of a ductile fracture model for three structural steels. Eng. Fract. Mech..

[67] Depraetere R., De Waele W., Cauwels M., Depover T., Verbeken K., Hertelé S. Modeling of Hydrogen-Charged Notched Tensile Tests of an X70 Pipeline Steel with a Hydrogen-Informed Gurson Model [Dataset]. https://osf.io/sak3w/.

[68] Tadashi K., Toshimitsu A.Y., Go O., Toshihito O., Manabu E. (2021). Modelling of hydrogen diffusion in a weld cold cracking test: Part 1, experimental determinations of apparent diffusion coefficient and boundary condition. ISIJ Int..

